# Supplementary material for: Suppression of flavivirus transmission from animal hosts to mosquitoes with a mosquito-delivered vaccine
Source: Nat Commun. 2022 Dec 16;13:7780. doi: 10.1038/s41467-022-35407-x (PMC9755785; doi:10.1038/s41467-022-35407-x)
Supplement: Supplementary file 1 — Supplementary Figures [file 41467_2022_35407_MOESM1_ESM.pdf]

Supplementary Information

Suppression of flavivirus transmission from animal hosts to mosquitoes with a mosquito-delivered vaccine

Dan Wen, Limin S. Ding, Yanan Zhang, Xiaoye Li, Xing Zhang, Fei Yuan, Tongbiao Zhao, Aihua Zheng

Correspondence to: zhengaihua@ioz.ac.cn.

This PDF file includes: Supplementary Figures 1-10

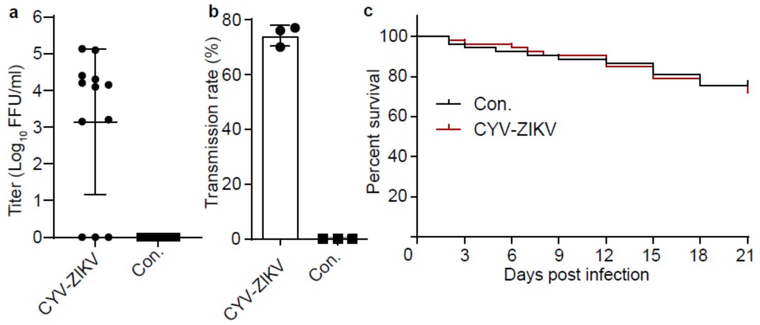

Supplementary Fig. 1. Survival of *A. aegypti* mosquitoes after infection with CYV-ZIKV. Five- to six-day-old females were blood-fed with CYV-ZIKV diluted to 1 × 10^8 FFU/ml. Control (Con.) is mock infected. (a) The viral titers in the saliva of a single mosquito were determined by the focus-forming assay with a 4G2 antibody at 12 dpi (n = 12). Data are presented as means ± SD. (b) The percentage of 12 mosquitoes tested for CYV-ZIKV positive in saliva at 12 dpi (n = 3). Data are presented as means ± SD of triplicate measurements. (c) Survival curve of 53 mosquitoes after infection with CYV-ZIKV. Similar results were obtained in three independent experiments. Source data are provided as a Source Data file.

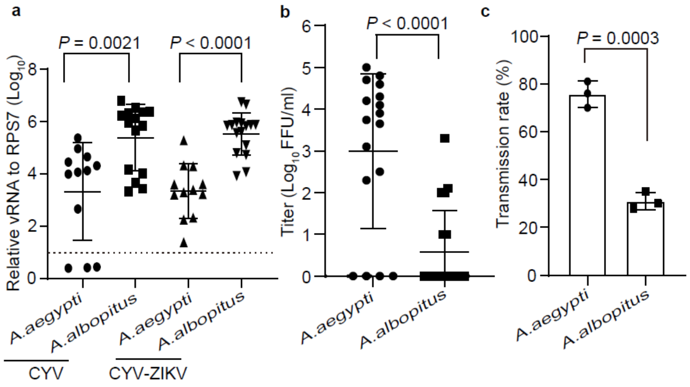

Supplementary Fig. 2. Susceptibility of CYV-ZIKV between *A. aegypti* and *A. albopictus*. Females were blood-fed with CYV-ZIKV or CYV diluted to 1×10^8 FFU/ml. One dot represents a mosquito. (a) The viral load of the whole individual mosquito (*A. aegypti*, n = 12; *A. albopictus*, n = 15) was detected by real-time PCR at 7 dpi. The viral RNA level was normalized against the reference gene RPS7. The *P*-value was determined by a two-sided multiple *t*-test. Data are presented as means ± SD. The dashed lines indicate the detection limit. (b) The titer of CYV-ZIKV in the saliva of a single mosquito was detected by the focus-forming assay at 12 dpi (n = 17). Data are presented as means ± SD. The *P*-value was determined by a two-sided unpaired *t*-test. (c) The percentage of 17 mosquitoes tested positive for CYV-ZIKV in their saliva (n = 3). Data are presented as means ± SD of triplicate measurements. The *P*-value was determined by a two-sided unpaired *t*-test. Similar results were obtained in three independent experiments. Source data are provided as a Source Data file.

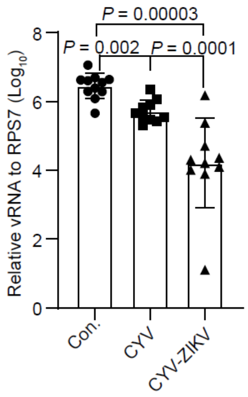

Supplementary Fig. 3. CYV-ZIKV suppresses ZIKV infection in *A. aegypti* mosquitoes. Mosquitoes were infected with ZIKV diluted to 10^5 FFU/ml by blood-feeding at 7 days post-blood feeding with CYV-ZIKV (n = 10), CYV (1 × 10^8 FFU/ml, n = 10), or PBS control (Con., n = 11). The ZIKV RNA level of a single mosquito was detected with real-time PCR at 7 dpi with ZIKV and normalized against the reference gene RPS7. Data are presented as means ± SD. The *P*-value was determined by a two-sided multiple *t*-test. Similar results were obtained in three independent experiments. Source data are provided as a Source Data file.

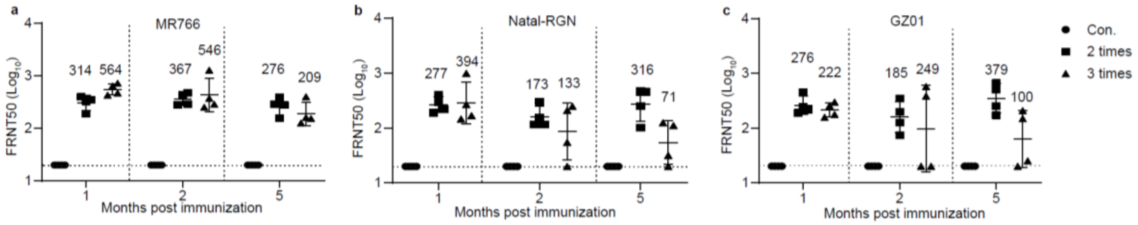

Supplementary Fig. 4. Persistence of humoral immune response by mosquito vaccine in IFNAR$^{-/-}$ C57BL/6 mice. IFNAR$^{-/-}$ C57BL/6 mice were immunized after being bitten by 10 CYV-ZIKV-mosquitoes two or three times, with an interval of 2 weeks (n = 4). The control group (Con.) was bitten by naïve mosquitoes three times. Neutralizing antibody titers were evaluated against the ZIKV MR766 (a), Natal-RGN (b), or GZ01 (c) strain using a FRNT50 assay at 1, 2, and 5 months after the last bite. The numbers for each group represent the mean neutralizing antibody titers. Data are presented as means ± SD. Similar results were obtained in two independent experiments. Source data are provided as a Source Data file.

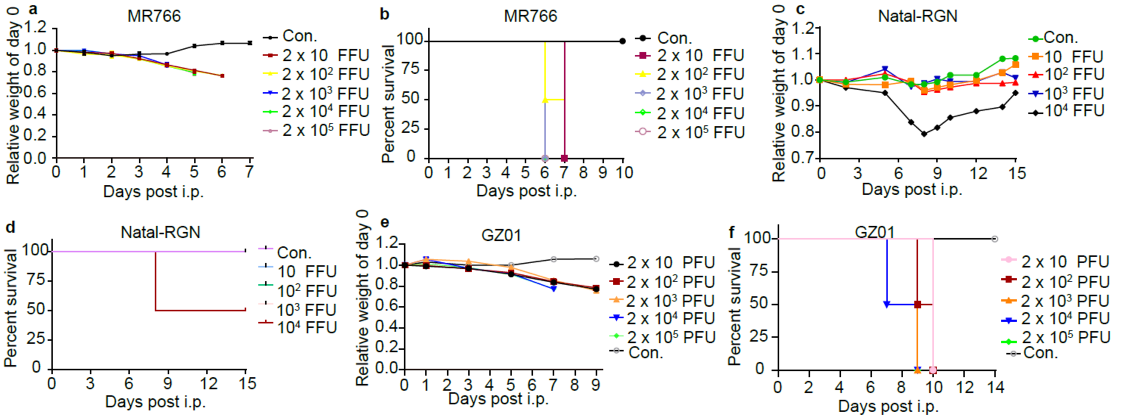

Supplementary Fig. 5. LD_{50} of various ZIKV strains in IFNAR$^{-/-}$ C57BL/6 mice. Mice (n = 2) were inoculated with the indicated doses of ZIKV MR766, Natal-RGN, or GZ01 via i.p. route. The control group (Con.) was inoculated with PBS via the i.p. route. Weight change (a, c, e) and survival rate (b, d, f) were monitored. Data are presented as means ± SD. Source data are provided as a Source Data file.

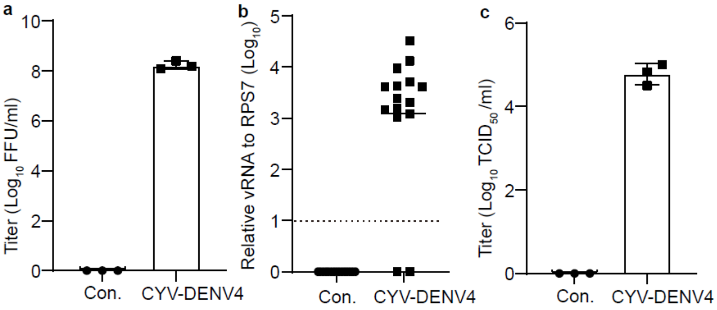

Supplementary Fig. 6. Susceptibility of CYV-DENV4 in *A. aegypti*. (a) Titer of CYV-DENV4 in the C6/36 supernatant at 4 dpi (MOI = 0.1). Con. indicates mock infected cells. Data are presented as means ± SD of triplicate measurements. n = 3. (b, c) Females were blood-fed with CYV-DENV4 diluted to 1×10^8 FFU/ml. (b) The viral RNA level of the whole individual mosquito was detected by real-time PCR at 7 dpi and normalized against the reference gene RPS7 (n = 15). Con. indicates mock infected mosquitoes. The horizonal bar indicates the mean. (c) The titer of CYV-DENV4 in the saliva of 80 mosquito was detected by $TCID_{50}$ at 12 dpi (n = 3). Con. indicates mock infected mosquitoes. Data are presented as means ± SD of triplicate measurements. Similar results were obtained in three independent experiments. Source data are provided as a Source Data file.

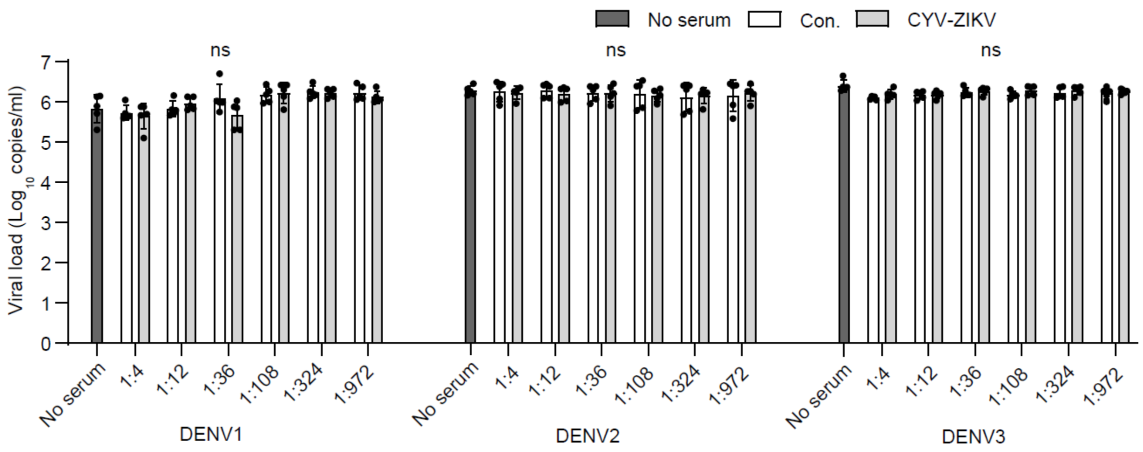

Supplementary Fig. 7. No ADE of DENV mediated by CYV-ZIKV immunized sera in K562 cells. The K562 cells were seeded in 24-well plates (10^6 cells/well). Mice sera (n = 5) were 3-fold serial diluted from 1:4 of in DMEM and incubated with equal volume of DENV1 West Pacific strain, DENV2 New Guinea C strain or DENV3 H87 strain (10^5 FFU) for 1 h at 37°C, then transferred to K562 cells. Viral RNAs in the supernatants were measured using real-time PCR at 4 dpi. Con. represents sera of naïve mice. Data are presented as means ± SD. The *P*-value was determined by a two-sided multiple *t*-test, and ns indicates not significant. Similar results were obtained in two independent experiments. Source data are provided as a Source Data file.

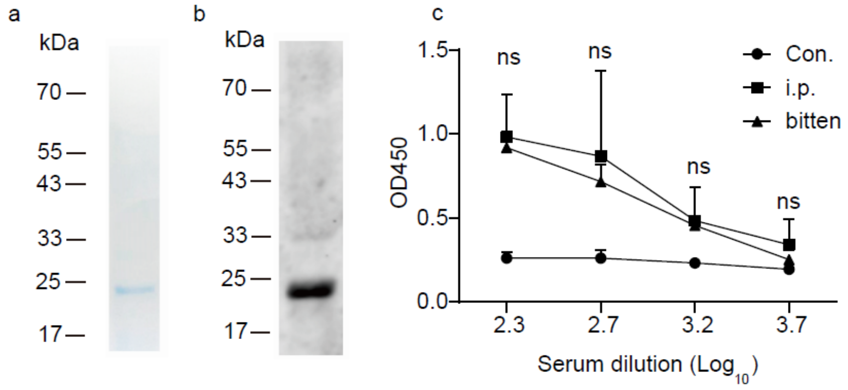

Supplementary Fig. 8. The level of ZIKV prM-specific antibodies in CYV-ZIKV-immunized mice by mosquito bites or via i.p. route. The purified ZIKV prM was detected by Coomassie Blue staining (a) and Western blot (b) by anti-His tag antibody diluted at 1: 5000 (Cat. MF082-HRP-01, Mei5bio, China). (c) The level of prM-specific antibody was detected by ELISA. Con. represents sera of naïve mice. i.p. indicates sera of mice received two dose vaccines via the i.p route. Bitten indicates sera of mice bitten by mosquitoes for three times. Con., n = 3; i.p., n = 6, bitten, n = 5. Data are presented as means ± SD. The statistical significance between i.p. and bitten group was determined by a two-sided multiple *t*-test, and ns indicates not significant. Similar results were obtained in three independent experiments. Source data are provided as a Source Data file.

Supplementary Figure 9

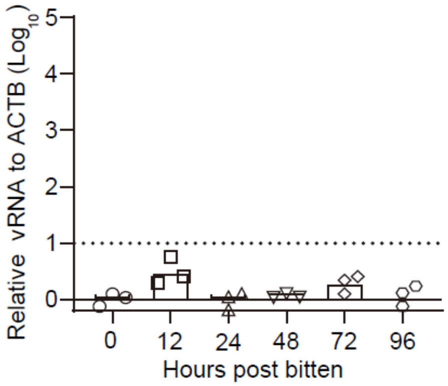

Supplementary Fig. 9. No replication of CYV-ZIKV in the local skin of mosquito bite site. IFNAR$^{-/-}$ C57/BL6 mice were anesthetized and 1 cm^2 of the abdominal hair was shaved while the surrounding area was covered with paper. The shaved skin was bitten by 30 CYV-ZIKV-carrying mosquitoes per mouse for 20 min. The skin tissues at the bitten site were dissected after scarification 12, 24, 48, 72, or 96 h post-bite (n = 3). The viral RNA of CYV-ZIKV in the skin was determined by real-time PCR using ACTB as the reference. The dashed lines indicate the detection limit. Source data are provided as a Source Data file.

Supplementary Figure 10

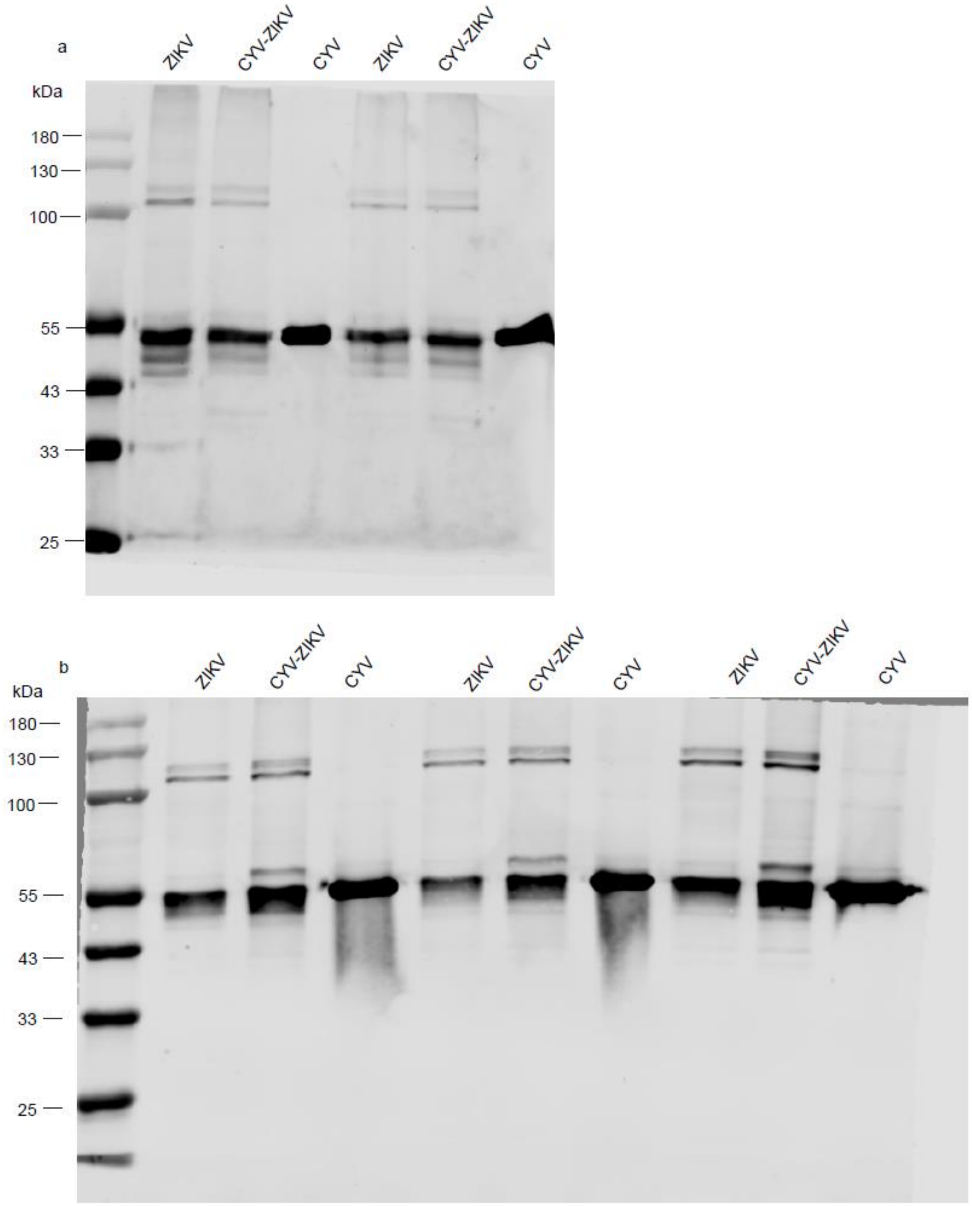

Supplementary Fig. 10. The raw data of Fig. 1c. a and b are two independent experiments. Source data are provided as a Source Data file.
