# Supplementary material for: Suppression of flavivirus transmission from animal hosts to mosquitoes with a mosquito-delivered vaccine
Source: Nat Commun. 2022 Dec 16;13:7780. doi: 10.1038/s41467-022-35407-x (PMC9755785; doi:10.1038/s41467-022-35407-x)
Supplement: Supplementary file 3 — Reporting Summary [file 41467_2022_35407_MOESM3_ESM.pdf]

nature portfolio

Corresponding author(s): Aihua Zheng

Last updated by author(s): Nov 20, 2022

Reporting Summary

Nature Portfolio wishes to improve the reproducibility of the work that we publish. This form provides structure for consistency and transparency in reporting. For further information on Nature Portfolio policies, see our Editorial Policies and the Editorial Policy Checklist.

Statistics

For all statistical analyses, confirm that the following items are present in the figure legend, table legend, main text, or Methods section.

n/a	Confirmed	
☐	☒	The exact sample size (*n*) for each experimental group/condition, given as a discrete number and unit of measurement
☐	☒	A statement on whether measurements were taken from distinct samples or whether the same sample was measured repeatedly
☐	☒	The statistical test(s) used AND whether they are one- or two-sided<br>*Only common tests should be described solely by name; describe more complex techniques in the Methods section.*
☒	☐	A description of all covariates tested
☐	☒	A description of any assumptions or corrections, such as tests of normality and adjustment for multiple comparisons
☐	☒	A full description of the statistical parameters including central tendency (e.g. means) or other basic estimates (e.g. regression coefficient) AND variation (e.g. standard deviation) or associated estimates of uncertainty (e.g. confidence intervals)
☐	☒	For null hypothesis testing, the test statistic (e.g. *F*, *t*, *r*) with confidence intervals, effect sizes, degrees of freedom and *P* value noted<br>*Give P values as exact values whenever suitable.*
☒	☐	For Bayesian analysis, information on the choice of priors and Markov chain Monte Carlo settings
☒	☐	For hierarchical and complex designs, identification of the appropriate level for tests and full reporting of outcomes
☒	☐	Estimates of effect sizes (e.g. Cohen's *d*, Pearson's *r*), indicating how they were calculated

Our web collection on statistics for biologists contains articles on many of the points above.

Software and code

Policy information about availability of computer code

Data collection	Fluorescent images data were collected from Zeiss Zen 2010 software (version 6.0; Zeiss, Germany) by Zeiss LSM 710 confocal microscopy (Carl Zeiss Meditec AG, Jena, Germany). RT-PCR data were collected from QuantStudio 12K Flex Software (version 1.4; ABI, USA) by ABI QuantStudio 12K Flex Real-Time PCR System (Applied Biosystems, Life Technologies, Carlsbad, CA, USA). Western blot images data were collected from Image Studio software (version 5.2; LI-COR, USA) by LI-COR Odyssey CLx Infrared Imaging System (LI-COR Biosciences, Lincoln, NE, USA). Absorbance values at 450 nm in ELISA assays were collected from SoftMax Pro 7 software (version 7.1; Molecular Devices, USA) by SpectraMax i3 multi-mode multiplate reader (Molecular Devices, San Jose, CA, USA).
Data analysis	Data were first collated using Microsoft Excel and then imported into GraphPad Prism 8 software (version 8.3.0; GraphPad software Inc, USA) to analyze statistical parameters including means, standard deviation, significant differences. Confocal images were analyzed using Zeiss Zen 2010 software (version 6.0; Zeiss, Germany).

For manuscripts utilizing custom algorithms or software that are central to the research but not yet described in published literature, software must be made available to editors and reviewers. We strongly encourage code deposition in a community repository (e.g. GitHub). See the Nature Portfolio guidelines for submitting code & software for further information.

Data

Policy information about availability of data

All manuscripts must include a data availability statement. This statement should provide the following information, where applicable:

- Accession codes, unique identifiers, or web links for publicly available datasets
- A description of any restrictions on data availability
- For clinical datasets or third party data, please ensure that the statement adheres to our policy

Sequences information were all acquired from NCBI database, including Chaoyang virus gene (Genbank sequence accession number: NC_017086), Zika virus MR766 strain sequence (Genbank sequence accession number: HQ234498), ZIKV Natal-RGN (Genbank sequence accession number: KU527068), ZIKV GZ01 (Genbank sequence accession number: KU820898), DENV1 West Pacific strain (Genbank sequence accession number: U88535), DENV2 New Guinea C strain (Genbank sequence accession number: M29095), DENV3 H87 strain (Genbank sequence accession number: KU050695) and DENV4 1228 strain (Genbank sequence accession number: KX239897). Plasmids used in the study were freely available upon request. No custom code or mathematical algorithm were used in this work. All data were available within this article, as well as supplementary information or source data files. All protocols have been described in Methods or in references therein.

Human research participants

Policy information about studies involving human research participants and Sex and Gender in Research.

Reporting on sex and gender	N/A
Population characteristics	N/A
Recruitment	N/A
Ethics oversight	N/A

Note that full information on the approval of the study protocol must also be provided in the manuscript.

Field-specific reporting

Please select the one below that is the best fit for your research. If you are not sure, read the appropriate sections before making your selection.

☒ Life sciences ☐ Behavioural & social sciences ☐ Ecological, evolutionary & environmental sciences

For a reference copy of the document with all sections, see nature.com/documents/nr-reporting-summary-flat.pdf

Life sciences study design

All studies must disclose on these points even when the disclosure is negative.

Sample size	Sample size for each experiment was described in figure legends and determined based on statistical analysis. Sample size was in respect with ethical criteria to use the minimal number of each experiment. Samples in mice and mosquito experiment were determined to get biological meaningful results. Simple sizes for real-time PCR, viral titer and neutralizing antibody were designed for at least three samples in each group for analysis.
Data exclusions	No data were excluded
Replication	All experiments were replicated three or more times. For immunization of mice via CYV-ZIKV-carrying mosquito bites, each group at least contained 5 mice. For measuring virus loading, each mosquito was collected and analyzed as an individual replicate and each group at least contained 10 mosquitoes in figure 6f .
Randomization	Mice and new adult insects were randomly allocated to the experiments.
Blinding	No blinding occurred during these studies. Experimental design did not require blinding because assessed variables are not confounded by the evaluator. We only focused on measurable variables (weight, number of eggs, survival rate, etc).

Reporting for specific materials, systems and methods

We require information from authors about some types of materials, experimental systems and methods used in many studies. Here, indicate whether each material, system or method listed is relevant to your study. If you are not sure if a list item applies to your research, read the appropriate section before selecting a response.

Materials & experimental systems

n/a	Involved in the study
☐	☒ Antibodies
☐	☒ Eukaryotic cell lines
☒	☐ Palaeontology and archaeology
☐	☒ Animals and other organisms
☒	☐ Clinical data
☒	☐ Dual use research of concern

Methods

n/a	Involved in the study
☒	☐ ChIP-seq
☒	☐ Flow cytometry
☒	☐ MRI-based neuroimaging

Antibodies

Antibodies used

The mouse monoclonal antibody 4G2 (hybridoma clone D1-4G2-4-15) was a commercial reagent (Cat. GTX57154, GeneTex, China), which recognizing fusion peptide of E protein among flaviviruses (1:120 dilution).
Goat anti-mouse IgG (H+L) cross-absorbed secondary antibody, Alexa Fluor 488 was purchased from Invitrogen (Cat # A-11001, InvitrogenTM, USA), which was used at a ratio of 1:400 in immunofluorescence assays.
M5 His-Tag (10E2) HRP conjugated Mouse monoclonal antibody (Cat. MF082-HRP-01, Mei5bio, China) was used at 1: 5000 to specifically recognized with prM protein in western blot assays.
HRP-conjugated Affinipure Goat anti-Mouse IgG (H+L) was purchased from Proteintech (Cat SA00001-1, Proteintech, USA) for ELISA assay at a ratio of 1:1000.

Validation

4G2 is a mouse monoclonal antibody that recognizes fusion peptide of E protein among all flaviviruses, including DENV and ZIKV. Commercial primary antibododies were validated by manufacturers, like Anti-Flavivirus group antigen from the hybridoma clone D1-4G2-4-15 (4G2), which was verified specific reactivity with DENV, WNV, YFV, ZIKV in publications (https://www.genetex.cn/Product/Detail/Flavivirus-group-antibody-D1-4G2-4-15-4G2/GTX57154). In addition, the 4G2 primary antibody used in our study was also validated in our previous work (doi:10.1073/pnas.2110491119).

Eukaryotic cell lines

Policy information about cell lines and Sex and Gender in Research

Cell line source(s)

All cell lines we used were from ATCC, the detailed information were as follows.
Vero cells we bred were from ATCC, CCL-81.
BHK-21 cells were from ATCC, CCL-10.
HEK293T cells were also from ATCC, CRL-3216.
Aedes albopictus clone C6/36 cells were from ATCC, CRL-1660.
K562 cells were from ATCC, CCL-243.

Authentication

All cell lines mentioned above were received authenticated from ATCC.

Mycoplasma contamination

All cell lines tested negative for mycoplasma contamination.

Commonly misidentified lines
(See ICLAC register)

No commonly misidentified lines.

Animals and other research organisms

Policy information about studies involving animals; ARRIVE guidelines recommended for reporting animal research, and Sex and Gender in Research

Laboratory animals

Aedes aegypti mosquitoes (UGAL/Rockefeller strain) and Aedes albopictus mosquitoes (Jiangsu strain) were bred at (26±1) ? and (75 ±5) % relative humidity conditions with a photoperiod of 14 h : 10 h of light : dark cycles. Larval mosquitoes were fed with the mixture of pulverizing mice food, albumin and yeast extract. Adults were fed on water and 10% (w/v) sucrose. For virus oral infection, five- to six-day-old female adults (Aedes aegypti or Aedes albopictus) were starved for 20 h prior to blooding meal with viruses. For intrathoracic microinfection, three-day-old adults (Aedes aegypti or Aedes albopictus) were intrathoracically microinjected with 60 FFU viruses.
Specific-pathogen-free C57BL/6 mice were purchased from Beijing Vital River Laboratory Animal Technology (licensed by Charles River) and bred for 2 weeks to acclimation. IFNAR-/- C57BL/6 mice were bought from the Institute Laboratory Animal Science, Chinese Academy of Medical Science & Peking Union Medical college. These mice were bred to 6-8 weeks old and immunized in the study.Mice were housed under the following conditions: ambient temperature 22±1?, humidity control 50%, 12 h light/12 h dark cycle.

Wild animals

No wild animals.

Reporting on sex

For mosquito feeding blood experiment, mosquito should be female. Mice immunization experiment includes male and female.

Field-collected samples

No field-collected samples.

Ethics oversight	All mice and mosquito experiments were performed strictly following bioethics principles and were supervised by the Bioethics Committee of the Institute of Zoology, Chinese Academy of Science.

Note that full information on the approval of the study protocol must also be provided in the manuscript.
